# Experience of healthcare among the homeless and vulnerably housed a qualitative study: opportunities for equity-oriented health care

**DOI:** 10.1186/s12939-019-1004-4

**Published:** 2019-07-01

**Authors:** Eva Purkey, Meredith MacKenzie

**Affiliations:** 1 Department of Family Medicine, Queen’s University, 220 Bagot street, Kingston, Ontario K7L 3G2 Canada; 2 Street Health Centre, a part of Kingston Community Health Centres, Kingston Ontario115 Barrack St, Kingston, Ontario K7L 3N6 Canada

**Keywords:** Homelessness, Health equity, Vulnerable populations

## Abstract

**Background:**

People experiencing homelessness are often marginalized and are known to face barriers to accessing acceptable and respectful healthcare services. This study examines the experience of accessing hospital-based services of persons experiencing homelessness or vulnerable housing in southeastern Ontario and considers the potential of Equity-Oriented Health Care (EOHC) as an approach to improving care.

**Methods:**

Focus groups and in-depth interviews with people with lived experience of homelessness (n=31), as well as in-depth interviews of health and social service provider key informants (n=10) were combined with qualitative data from a survey of health and social service providers (n=136). Interview transcripts and written survey responses were analyzed using directed content analysis to examine experiences of people with lived experience of homelessness within the healthcare system.

**Results:**

Healthcare services were experienced as stigmatizing and shaming particularly for patients with concurrent substance use. These negative experiences could lead to avoidance or abandonment of care. Despite supposed universality, participants felt that the healthcare system was not accountable to them or to other equity-seeking populations. Participants identified a system that was inflexible, designed for a perceived middle-class population, and that failed to take into account the needs and realities of equity-seeking groups. Finally, participants did identify positive healthcare interactions, highlighting the importance of care delivered with dignity, trust, and compassion.

**Conclusions:**

The experiences of healthcare services among the homeless and vulnerably housed do not meet the standards of universally accessible patient-centered care. EOHC could provide a framework for changes to the healthcare system, creating a system that is more trauma-informed, equity-enhancing, and accessible to people experiencing homelessness, thus limiting identified barriers and negative experiences of care.

**Electronic supplementary material:**

The online version of this article (10.1186/s12939-019-1004-4) contains supplementary material, which is available to authorized users.

## Background

This study explores the experience of hospital-based healthcare for people who are vulnerably housed or homeless. Literature suggests that the healthcare system is either inaccessible to or fails to meet the needs of certain groups. Data outlines barriers to care for Indigenous Canadians, members of the LGTBQ* community, persons experiencing ongoing or historical trauma, persons using substances, and those experiencing homelessness or who are vulnerably housed [1–10]. Thirty-five thousand Canadians are homeless on any given night and 235,000 Canadians experience homelessness in a year [11]. Average life expectancy for homeless persons is estimated at between 42 and 52 years [12, 13]. Between 44 and 60% of people who experience homelessness will use illicit substances in their lifetime [11, 14, 15].

The primary objective of the Canada Health Act, the foundational legislation of Canada’s universal healthcare system, is “to protect, promote and restore the physical and mental well-being of residents of Canada and to facilitate reasonable access to health services without financial or other barriers” [16]. This would imply that health services must be tailored to eliminate avoidable barriers to access, and should actively seek to protect, promote and restore the health of all Canadians, including the most marginalized.

Data in this study derives from a mixed-methods study funded by the South East Local Health Integration Network (SELHIN) (Ontario, Canada) exploring palliative care services for the homeless and vulnerably housed. In this study, “homelessness” or “vulnerably housed” includes those who are living out-of-doors, in substandard conditions not fit for human habitation, in temporary or unstable accommodations, in shelters, and those who are at risk of losing their existing housing [17].

## Methods

### Study design

**Fig. 1:**
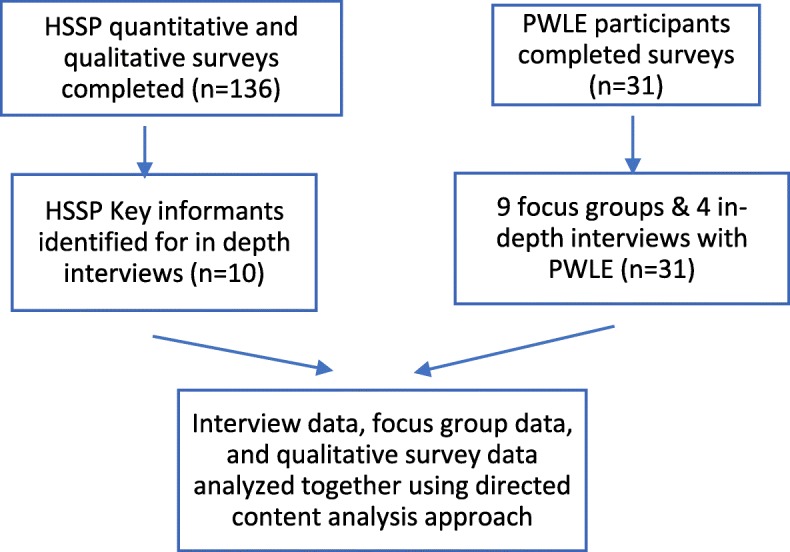
Data collection

A survey was used to obtain data from health and social services providers (HSSPs) and interviews were conducted with key informants (KIs) from this group. A survey along with focus groups and in-depth interviews collected data from participants with lived experience of homelessness. See Fig. 1 for an outline of all data collection and Additional file 1 for survey tools and interview guides. Ethics approval was obtained through Queen’s University Health Sciences and Affiliated Teaching Hospitals Research Ethics Board.

### Participants and sampling.

#### Health and social service providers (HSSPs)

A survey was distributed by email widely to organizations throughout the SELHIN who work with people experiencing homelessness (mental health and addiction agencies, housing agencies, legal aid, shelters, food programs, community health centres, primary and palliative care providers among others). The survey included questions about the participant’s organization, scope of practice, and thoughts and opinions on the provision of care to people experiencing homelessness with an explicit emphasis on end of life care. All questions were multiple choice, however they all had free text spaces in which participants could include comments and other considerations. 136 HSSPs responded to the survey.

Following survey collection, community agencies identified KIs who had been employed by the organization for at least 1 year and had provided front line services (past or current). KIs were a mix of urban, rural and semi-rural. Research assistants conducted ten in-person or telephone interviews using a semi-structured interview guide exploring themes that were developed following review of the survey findings: challenges in accessing palliative care services by people experiencing homelessness; the impact of substance use on service acceptability and access; education and resources required to support people experiencing homelessness and/or substance use; and recommendations for system changes. Interviews lasted approximately 60 min and were conducted by research assistants or by the principle investigator (MM).

#### Persons with lived experience (PWLE)

Persons with experience of homelessness were recruited from agencies mandated to provide services to those who are vulnerably housed. Snowball sampling was used to recruit further participants. Inclusion criteria was a past or present history of homelessness. Participants completed a survey collecting basic demographic variables, information on education, housing, service use, self-rated mental and physical health, and substance use. Support was provided when literacy was a concern. Six focus groups were held with 2 to 7 participants, along with 4 in-depth interviews, all lead by research assistants or by the principle investigator, most of whom had experience working with people with lived experience of poverty and substance use. The intent was to run focus groups exclusively, but due to scheduling challenges, several participants were interviewed individually. Both interviews and focus groups used the same semi-structured interview guide exploring themes related to how homelessness affects access to palliative care, the impact of substance use, thoughts on death and dying, and recommendations to improve end of life care for vulnerably house people. Theme saturation was reached prior to the end of the data collection period in that no new themes were appearing in the interviews and focus groups, however due to the importance of including the community in the research process, the desire to have a large number of participants with lived experience, and the fact that other participants had already been recruited and expected to participate, two final focus groups were completed. 31 people participated, the sessions lasted between 2 and 3 h and were held at Street Health Centre in Kingston. Participants were given a stipend of 50$ for their participation.

### Data Analysis.

KI interviews and focus groups were audio-recorded and transcribed. HSSP surveys free text responses provided sufficiently rich written content to include with the transcripts. While initially reviewed for findings relevant to palliative care, transcripts were reviewed a second time by both researchers seeking themes related to access to and experience of healthcare services in general. Transcripts and survey responses were reviewed, analyzed and coded as one data set by 2 independent researchers, both of whom have experience with vulnerable populations as well as research and clinical experience in trauma and violence informed and EOHC approaches. Themes were then reviewed collaboratively by the 2 researchers to ensure consensus. The analysis was informed by directed content analysis [18, 19] which can be used when there is existing theory about a phenomenon, but this theory is incomplete. It is notable that for the PWLE, very few had had first-hand personal experience with end of life care, and much of their discussion related to the provision and receipt of healthcare services more broadly. Data was sufficiently rich that there were extensive findings related to healthcare experiences in general, and that theme saturation was felt to be reached for these themes despite this not having been the original focus of the project.

## Results

Quantitative data will be reported in detail elsewhere [20, 21]. Sociodemographic data relevant to the discussion is included in Table 1.

**Table 1:** Participant Organizational and Socio-demographic data

Variable	Frequency (%)
Health and Social Service Providers:	
Organization provides care to persons experiencing homelessness or unstable housing	74%
Organization provides care to persons experiencing substance use	89%
People with lived experience of homelessness	
Identifying as First Nations, Inuit or Metis	13%
Completed high school of higher education	68%
Minimum of part time employment	33%
Duration of homelessness months or years	39%
Frequency of homelessness	
Once	100%
> 5 times	17%
Service use (last 6 months):	
Emergency department	55%
Healthcare clinic	48%
Hospital	45%
Ambulance services	19%
Self-reported mental health average or poor	58%
Substance use (last 3 months):	
Alcohol	68%
Benzodiazepines	
Cocaine	32%
Opioids	29%
Methadone or Buprenorphine/naloxone	26%
Crystal Methamphetamine	45%

### Qualitative findings

Participants’ experience of either accessing services themselves, or of assisting clients to access services, was predominantly negative. Four themes were highlighted by participants: (1) experiences and consequences of stigma and shame when accessing healthcare; (2) lack of accountability of the healthcare system towards equity seeking populations; (3) inflexibility of the healthcare system; and (4) positive experiences that warrant discussion for what they teach us about potential improvements.

### Experience of stigma and shame when interfacing with the healthcare system

The experiences of stigma among PWLE were overwhelming. In some cases, the stigma was so painful that it superseded any health complaints, previous trauma, or other concerns a patient might have. Stigma was by far most pronounced in the context of current or documented history of substance use, even if substance was remote, compounding the sense of shame and stereotyping.

**Table:** 

Box 1 Participant Quote *I’ll share a story that was shared with me. [This woman] had suffered a brutal rape. Horrific. Absolutely horrific. She was a woman in her late 40s. Lived on the street throughout her whole life, back and forth. She was telling me her story. She needed to share it […]* *and she didn’t cry. Not one tear when she talked about the abuse that she endured [..]. She wept when she talked about how she got treated at the hospital because she was bleeding so profusely and she flinched at a needle and the comment from the nurse was made to her: 'Well look at your arms, as if you have a problem with needles". That weighed so heavily and this is when the woman broke down. The abuse was horrific but she had almost been marinating in that level of violence and abuse all her life. The devastating part of it all was the shame she felt from the hospital […]* *because of her I.V. drug use. (KI 4).*	

Stigma, or anticipated stigma, had important consequences for health. As reported by others [22], PWLE avoid care due to past negative experiences. They might leave in the middle of a care session, even removing intravenous lines in order to extricate themselves from intolerably stigmatizing situations. PWLE were often isolated from support networks when in care because their support networks, coming from the same social contexts, were equally stigmatized and occasionally overtly excluded by healthcare providers. PWLE lacked trust towards healthcare providers due to past experiences which had significant impacts on their care seeking behavior and likelihood of following through on provider recommendations. Finally, PWLE and HSSPs had many examples in which they felt that complaints were not taken seriously, often due to a history of substance use, which caused them to fear that they would be unable to obtain appropriate care.

**Table:** 

Box 2 Participant Quote *It actually got so bad that I actually unhooked my IV and left the hospital and didn’t go back. […]* *I just couldn’t. believe it. It was scary actually because when I unhooked that IV, I thought to myself: ‘What am I doing here?’ That’s how scared I was that they actually set it off in me that I started to think ‘Oh god, now they are going to do this to me and now they’re not gonna take proper care of me.’ (FG1A).*	

The presence of an advocate from outside of their social network (eg. a social services worker or pastoral care worker), had a significant impact on the care patients received. While this was more likely to enable them to receive care in a respectful and appropriate manner, it further highlighted the stigma they experienced when their advocate was not present.

### Lack of accountability of the healthcare system towards equity-seeking populations

Participants felt that the healthcare system was not accountable to the people it served. Participants articulated the responsibility of healthcare providers to provide excellent, empathic care to *everyone* who presents, regardless of their socioeconomic status, substance use history, or life circumstances. Healthcare providers were felt to have a lack of understanding of the impact of social determinants of health, ongoing trauma and past adversity on people’s health and healthcare presentations. Examples were given of clients asked to leave the hospital because of the way they dressed or smelled. Participants felt that healthcare providers lacked knowledge around harm reduction, around the root causes of substance use and adversity, and that they appeared to lack empathic or compassionate curiosity towards patients and the difficulties they encountered.

**Table:** 

Box 3 Participant Quotes *“Being homeless- I mean people look at you as though you’re a low life, piece of crap. I mean, you’re a drug addict and everything else. You’re not worth the shit that you sleep in [..]. You’re restricted because of the way you look. You’re on the street. You don’t have a place. Doors are shut. People just shun you and everything else” (FG9B).* *“You know it’s all those kids we think about when we hear these horrific news stories of abuse. They went into the foster care system and then we don’t think of them again but that little kid ends up being the 30-year-old. with a criminal record and that little kid ends up being a woman who’s prostituted for the last 10 years.” (KI4).*	

Respondents wanted to see medical practitioners whose priority were their patients rather than status, job security, or finances. They also felt that having peers with lived experience of substance use, homelessness, or other equity-related challenges operating within the healthcare system would help make care more accountable and acceptable for them and others.

**Table:** 

Box 4 Participant Quote *“If you’re going to bring new [healthcare providers] in, then you educate them to be this way and if you. don’t treat this way. […]* *I mean - there’s a suggestion box [..]! You’re going to stand accountable. Let’s get the government accountable. Let’s get everybody accountable who’s looking after us. I AM a human being. If you’re not gonna to treat me like a human being - well you’re going to hear it right from me.” (FG1B).*	

### Inflexibility of the system

HSSPs described a healthcare system that was not tailored to meet the needs of their clients. The system was described as designed by middle class people for middle class clients, expecting conformity to the system rather than tailoring the system to the differing needs, desires and challenges of patients. Examples included the requirement that housing be obtained before treatment could be initiated when housing was not an option; a lack of flexibility for patients who might show up late or miss appointments; and a lack of openness to a harm reduction approach that might allow patients to receive a tailored form of treatment in the context of substance use rather than being dismissed out of hand.

### Positive experiences

While the majority of the discussion, both from HSSPs and PWLE, focused on negative experiences of care, there were also some positive encounters related to healthcare experiences where providers upheld dignity, autonomy and choice for patients, where they provided flexible, non-judgemental services in spaces where clients felt welcomed. Participants used terms such as “trust” and “compassionate” to describe these positive experiences of care.

**Table:** 

Box 5 Participant Quotes *“She’s a nurse here yes. I adore her. I adore her. I respect her and I trust her and she’s the sweetest girl that.* *I’ve ever had – the sweetest medical care person I’ve ever had take care of me. She’s just amazing […]* *Yeah.* *like she’s very very thorough and she’s very compassionate. I just, oh my heart’s with her, I love her. Yeah.”* *(FG9BRM1)* *“They are really like, hey we like the atmosphere of this place. We like that people here treat us really nice and we’re people. We feel loved. There are paramedics here who are, you know, assisting us. Um we really feel safe in this space and like there’s no judgement and we want to keep coming back here.” (KI9).*	

## Discussion

Our findings echo the negative experiences and resulting impacts on health and healthcare access of equity seeking populations described in other studies, including the homeless and vulnerably housed [1–8, 10, 23]. These include care avoidance, stigma, inflexibility of the current system, unmet healthcare needs and a lack of harm reduction philosophies integrated into the delivery of care.

While listening to the voices of our participants is key to understanding the inadequacies of our system, listening to these voices also presents an opportunity for change. There is small and increasing body of literature on Equity-Oriented Health Care (EOHC) and trauma and violence informed care in healthcare settings but these theories are rarely applied to hospital-based medicine and do not address hospital-based medicine for the homeless or vulnerably housed. We believe that the articulation of EOHC [24–26] as an approach may present us with a road map and tools to respond to the concerns of homeless and vulnerably housed clients, particularly with respect to their concerns about discrimination, stigma, and inflexibility of the system as articulated in our study and others [23, 27].

EOHC rests on 3 components. The first is trauma and violence informed care (TVIC) that recognizes the prevalence of past and ongoing trauma in people’s lives and acknowledges the way in which trauma affects people’s physical and emotional health, interpersonal relationships, and ability to access care. TVIC rests on 5 principles [28]: [1] Trauma awareness and acknowledgement; [2] Safety and trustworthiness; [3] Choice, control and collaboration; [4] Strengths based and skills building; and [5] Cultural, historical, and gender issues. The principles of TVIC are echoed in participants’ narratives. Participants shared the great burden of past and ongoing trauma that people facing homelessness and substance use have experienced. The need for safety and trust were explicitly articulated, as well as the challenges in developing that trust. Choice, control and collaboration are the antithesis of the stigmatizing and dismissive care that participants too often received in healthcare encounters, which is neither strengths based nor skills building. Finally, much literature supports the ongoing impact of gender, ethnicity, indigeneity and history on access to care [4, 6, 8].

The second component of EOHC is harm reduction. Most of the literature examining PWLE of homelessness identify substance use and the healthcare system’s response to substance use, as significant concerns [2, 14, 15, 23]. Harm reduction encompasses programs, practices, policies and philosophies that aim to reduce the harms of substance use, viewing substance use as a health issue rather than a moral failure [26]. Participants feel that healthcare providers view their substance use as making them less worthy of dignified care and less valuable as human beings. A harm reduction approach requires a fundamental shift in how the healthcare system interacts with people who use substances. In addition to formal policies and programs, such an approach requires us to see the *people* behind the substance use, to recognize their dignity, experiences, trajectories, and challenges.

Cultural safety is the third component of EHOC. Culturally safe care is particularly important in the Canadian context where Indigenous people continue to experience the negative effects of current and past colonization [6, 29] but would be relevant in any context of human diversity. Culturally safe care explicitly addresses inequitable power relations, racism, discrimination, and effects of historical and current inequities within health care encounters [29]. More than just an attitude, culturally safe care requires knowledge of history and of the root causes and consequences of inequity on the part of healthcare providers.

Finally, EOHC requires that an approach to and delivery of care be developed with input from all stakeholders, including people with lived experience, but also all members of the healthcare team from physicians and nurses to janitors and receptionists. A recent study has found that cross sector collaboration that provides integrated health care improved barriers to access and also enabled self-managed care [30]. These changes require leaders to engage not only with providers who are already advocates for equity-seeking populations, but also with those who are not. EOHC presents a unique opportunity to build partnerships among professional and patient groups that rarely mix outside of clinical care and allows a system to be responsive to the local needs of its population. Communities with higher rates of substance use, higher percentages of Indigenous clients, or recent loss of employment with increase in precarious housing could meet the challenges and opportunities presented by EOHC differently.

### Limitations

The HSSPs in our study were almost all involved in providing care to homeless and vulnerably housed individuals and were generally self-described advocates for this group. Our study might have benefitted from integrating the voices of HSSPs who are not specifically committed to working with equity-seeking populations. Additionally, our data was originally collected in the context of work on palliative care. Further questions specifically targeting other healthcare experiences might have yielded additional information. Nevertheless, our findings are amply supported by the extensive verbal and written discussions around healthcare services from all study participants and align with findings in the literature as well [1–3, 10, 23].

## Conclusions

There are two key messages in our findings: The first is that the care we are providing to our most vulnerable clients is not adequate and does not meet the professional standards of accessibility, universality, and patient-centeredness. An often-quoted line by Dr. Edward Trudeau from the 1800s proposes that the physician’s role is “to cure sometimes, to relieve often, to comfort always”. Our findings demonstrate that for certain groups we may be failing on all three counts.

Our second message is that we believe there is a way to raise our healthcare system to this standard, and that EOHC, developed locally and tailored to place, provides a road map from where we can begin. EOHC requires a cultural shift within our profession, away from the standardized one-size-fits-all care we have become used to and back, perhaps, to a more versatile, creative way of delivering care that many of us aspire to. It will require team work in hospitals and clinics, changes to curriculum in medical and nursing schools and continuing professional development. It will require those who hope to be leaders in this field to have compassion and understanding for colleagues for whom this is more difficult. Finally, it will require us to not only listen to, but to *hear* and to *see* the patients before us in all their strength, complexity and occasional despair, to consider the trajectory and meaning of their lives within our broader society, as well as our own privileged place therein.

## Additional file

Additional file 1: **Appendix A.** Health Care and Service Providers Survey. **Appendix B.** Participant with lived experience Survey. **Appendix C.** PWLE focus group and interview guide. **Appendix D.** KI Interview guide. (DOCX 26 kb)

## Data Availability

All transcripts of interviews and focus groups, survey responses, and quantitative data are available from the authors upon request. Quantitative data is currently under consideration for publication elsewhere.

## Abbreviations

EOHC: Equity-Oriented Health Care
HSSP: Health and social service provider
KI: Key information
SELHIN: South East Local Health Integration Network
TVIC: Trauma and Violence-Informed Care
