# Supplementary material for: Experience of healthcare among the homeless and vulnerably housed a qualitative study: opportunities for equity-oriented health care
Source: Int J Equity Health. 2019 Jul 1;18:101. doi: 10.1186/s12939-019-1004-4 (PMC6604349; doi:10.1186/s12939-019-1004-4)
Supplement: Supplementary file 1 — Appendix A. Health Care and Service Providers Survey. Appendix B. Participant with lived experience Survey. Appendix C. PWLE focus group and interview guide. Appendix D. KI Interview guide. (DOCX 26 kb) [file 12939_2019_1004_MOESM1_ESM.docx]

Additional file 1

Appendix A:

Health Care and Service Providers Survey

Thank you for agreeing to participate in this survey. Your feedback is important! Your answers will be kept confidential. Participation is voluntary–you can skip a question or stop the survey at any time you want.

1. Which community do you work and?
   1. Urban
   2. Rural
   3. Semirural
2. Which are the following best describes your organization’s area(s) of work (please check all that apply)?
   1. Health care
   2. Public health
   3. Harm reduction services
   4. Food assistance
   5. Housing assistance
   6. Social services
   7. Family and children services
   8. Community development
   9. Other (please specify)
3. Does your organization provide services to any of the following weaning vulnerable populations? Please note that homelessness refers to time spent at shelters, in a car, at a friends/family place or on the street because in individual does not have a place of their own.
   1. Homeless
   2. Persons with substance use disorders
   3. Indigenous peoples
   4. Veterans
   5. Other (please specify)
4. What is your current position at your organization?
5. How long have you been in your current position?
   1. Years__________
   2. Months________
6. Which are the following best describes your area(s) of work? (Please check all that apply)
   1. Health care
   2. Public health
   3. Harm reduction
   4. Food assistance
   5. Housing assistance
   6. Social services
   7. Family children services
   8. Community development
   9. First responder
   10. Other(please specify)
7. How do identify?
   1. Female
   2. Male
   3. Transgender female
   4. Transgender male
   5. Prefer not to answer
   6. Other (please specify)
8. Within the course of your work, how frequently are you in contact with homeless individuals?
   1. Never
   2. Daily
   3. Weekly
   4. Monthly
   5. Yearly
   6. I don’t know
   7. Other(please specify)
9. If your patients/clients require end-of-life/palliative care, what services are available to them in your community? (Please check all that apply)
   1. Home visits
   2. Hospital inpatient
   3. Hospital outpatient
   4. Residential hospice
   5. Respite
   6. I don’t know
   7. Other (please specify)
10. Do think people who are experiencing homelessness have difficulty accessing end-of-life/palliative care services?
    1. Yes, if yes please explain how
    2. No
    3. I don’t know
11. Do you think there are barriers to providing end-of-life/palliative care to a person who uses illicit substances?
    1. Yes, please specify the barriers/challenges
    2. No
    3. I don’t know

*For questions 12-13: thinking about a homeless client/patient who uses illicit substances:*

1. Do you think that your client’s/patient’s drug use affects their access to end-of-life care/palliative care?
   1. Yes, if yes, please specify how
   2. No
   3. I don’t know
2. Do you think that expanding harm reduction policies to include on-site, supervised drug use would encourage an individual using illicit substances to engage in end-of-life/palliative care?
   1. Yes, if yes please explain why
   2. No, if no please explain why not
3. Does your organization provide end-of-life/palliative care services?
   1. Yes
   2. No
   3. I don’t know

If participant’s organization provides palliative care services, survey will proceed to Q 15

If participant’s organization does not, survey will end

1. Does your organization provide end-of-life/palliative care at any of the following vulnerable populations? (Please check all that apply)
   1. Homeless
   2. Person with substance use disorder
   3. Indigenous peoples
   4. Veterans
   5. Other(please specify)
2. Which of the following end-of-life/palliative care services does your organization provide? (Please check all that apply)
   1. Advanced care planning
   2. Caregiver support
   3. case management/patient navigator
   4. Emotional/psychosocial support
   5. Medical management
   6. Spiritual support
   7. Other (please specify)
3. Where does your organization provide your patients/clients with end-of-life/palliative care services?
   1. Home
   2. Hospital inpatient
   3. Hospital outpatient
   4. Residential hospice
   5. Respite
   6. Clinic/office
   7. Other (please specify)
4. Who performed the initial end-of-life/palliative care consult visits? (Please check all that apply)
   1. Advanced practice clinicians (NP, RN,etc)
   2. Chaplain/spiritual care provider
   3. Case manager
   4. Licensed/registered practical nurse
   5. Physician
   6. Social worker
   7. Other(please specify)
5. How many end-of-life/palliative care consult visits dig her team completely in 2017?
   1. 0-5
   2. 6-10
   3. 11-20
   4. 21-30
   5. 30+
6. Are there environments where you feel you cannot provide end-of-life/palliative care?
   1. Yes, please detail
   2. No
7. Do you feel you have adequate training/resources to provide end-of-life care to a person experiencing homelessness?
   1. Yes
   2. No, please explain
8. Do you feel you have adequate training/resources to provide end-of-life care to a person using illicit substances?
   1. Yes
   2. No, please explain
9. Would your management change during end-of-life/palliative care if you knew your patient was using illicit substances?
   1. Yes, please describe how
   2. No
   3. I don’t know

Appendix B

Participant with lived experience Survey

1. What community do you live in?
2. How old are you
   1. <21
   2. 21-29
   3. 30-39
   4. 40-49
   5. 50-59
   6. 60-69
   7. 70+
3. How do you identify?
   1. Female
   2. Male
   3. Transgender female
   4. Transgender male
   5. Prefer not to answer
   6. Other (please specify)
4. Are you First Nations, Metis or Inuit person?
   1. Yes
   2. No
   3. Other (please specify)
5. Please described you’re race/ethnicity.
6. What is the highest level of school to you have completed?
   1. < 8^th^ grade
   2. Some high school
   3. High school diploma
   4. Some College/University
   5. College/University degree
   6. Other(please specify)
7. Have used any of the following services in the past 6 months?(Please check all that apply)
   1. Detox
   2. Rehab
   3. Drop- in spaces
   4. Food banks
   5. Harm reduction services
   6. Health clinics
   7. Emergency departments
   8. Hospitals
   9. Housing supports
   10. Job training/job supports
   11. Legal clinic
   12. Services to help you get ID
   13. Shelters
   14. Soup kitchens
   15. Other(please specify)
8. Have you had contact with any of the following in the past 6 months (please check all that apply)
   1. Police
   2. Ambulance
   3. Probation/parole
   4. Jail/detention Center
9. Where will you be sleeping tonight? (Please check box)
   1. Own home/apartment
   2. Family/friend’s home
   3. Shelter
   4. Vehicle
   5. Outside
   6. I don’t know
   7. Other(please specify)
10. Is this year first time being homeless?
    1. Yes
    2. No
    3. Not applicable
11. During the last 12 months, how many weeks or months have you been homeless? This includes time spent in shelters, on the Street, or elsewhere, or staying with other people because he did not have a place of your own. Please write the number:
    1. Weeks__________
    2. Months _________
12. How many times a day been homeless in the past and for how long each time (in weeks or months)?
    1. Once; weeks _______; months_______
    2. Twice
    3. 3 times
    4. 4 times
    5. 5 times
    6. More than 5 times
    7. Not sure
13. How long has it been since she last had a permanent place to stay? (Please fill in the number)
    1. Days
    2. Weeks
    3. Months
    4. Years
14. What you think is stopping you from having permanent housing rate now? (Please check all that apply)
    1. Unemployment
    2. Not enough income
    3. Rents are too high
    4. Family/relationship conflict/breakdown
    5. Eviction
    6. Mental health problem
    7. Physical health problem, including disability
    8. I don’t want housing
    9. Other(please specify)
15. If you are not seeking housing please list the reasons for not wanting it
16. Are you currently on a wait list for housing?
    1. Yes
    2. No
    3. I don’t know
17. Are you currently on Ontario Disability Support Program (ODSP)?
    1. Yes
    2. No
18. What sources of income to you have?(Please check all that apply)
    1. Full-time employment
    2. Part-time employment
    3. Seasonal employment
    4. Informal employment (eg., Side jobs here and there)
    5. ODSP
    6. Panhandling
    7. Investment income
    8. Ontario works
    9. Employment insurance
    10. Canada pension plan
    11. Guaranteed income supplement
    12. Old-age security
    13. Other (please specify)
19. Do you currently have any of the following health-related issues? (Please check all that apply)
    1. Asthma
    2. COPD
    3. Cancer
    4. Diabetes
    5. Heart disease
    6. Liver disease
    7. Addiction
    8. Mental health condition
    9. Physical disability
    10. Other(please specify)
20. Overall, how would you rate your mental health?
    1. Excellent
    2. Good
    3. Average
    4. Poor
    5. Not sure
21. Overall, how would you read your physical health?
    1. Excellent
    2. Good
    3. Average
    4. Poor
    5. Not sure
22. Have you used any of the following substances without a prescription in the last 3 months? (Please check all that apply)
    1. Alcohol
    2. Benzodiazepines
    3. Cocaine
    4. Crystal meth
    5. Amphetamine/stimulants (eg., Adderall, Vyvanse)
    6. Opioids (heroin, morphine, hydromorphone, fentanyl, etc)
    7. Marijuana
    8. Methadone/Suboxone
    9. Crack
    10. Other(please specify)
23. If you are using substances, has your substance use changed in the past year? (Please check all that apply):
    1. Using more
    2. Using less
    3. Using different drugs
    4. No difference
    5. Not applicable
    6. Other(please specify)
24. Please described why your substance use has changed

Appendix C

PWLE focus group and interview guide

| Time | Question/Topic | Prompts |
| --- | --- | --- |
| WARM UP QUESTION (10 minutes): Experience with death/dying | | |
| 10 minutes  10:15 -10:25 | Do you have any experience with a friend or relative who has a serious illness or injury or who has died? | - What was their experience like?   Is this an experience you would want?   - How could it have been different, what would have made it better? |
| THEME 1: How Homelessness may affect access to Palliative Care (20 minutes) PRIORITY QUESTION | | |
| 10:25 – 10:45  (10) | Do you think being homeless or vulnerably housed affects a person’s access to palliative care? | In what way(s) |
| (10) | Have you had a friend or family member with housing issues die? | - What was their experience like? - Is this an experience you would want? - How could it have been different? - What would have made it better? |
| THEME 2. Does Substance Use affect access to palliative care? (15 minutes) PRIORITY QUESTION | | |
| 10:45-11:00  (8) | If you use substances, are you worried how being in a traditional setting (eg hospital) will impact the care you receive at the end of your life? | - How likely are you to stay in care if your substance use isn’t accommodated? |
| (7) | What would good end of life care look like for a person who uses substances? | - Describe the setting? - Would being able to use substances be important? |
| Theme 3: Recommendations (15 minutes): What stands in the way of a good death for vulnerably housed people? PRIORITY QUESTION | | |
| 5 minutes  11:30-11:45 | What stands in the way of a good death? | - What stands in the way of good health care? - What would you need to die with comfort and dignity - What are some problems with services you have encountered? |
|  | What kind of services do you think are needed so that homeless people might die with comfort and dignity? |  |
| THEME 4: Thoughts on death and dying? (15-30minutes) | | |
| 11:00 – 11:30  5 | Do you think about death and dying? | - Is this an issue that concerns you - Is this an issue that you would like to talk about? |
| 10 | What concerns do you have about dying, end of life and death? | - Are you concerned about pain management? - Are you concerned about life support (being stuck on it)? - What are you afraid of when dying - Are you concerned about where you will die - Are you concerned about what happens to your body? |
| 5 | If you are sick and dying, are there people you trust/love that you can turn to for supports? | - Do you have family/friends you can count on? - Do you have people you trust to make decisions for you? - Are there any health care or social service providers you can rely on? |
| 10 | Can you describe a good death? | - Where would you like to die - Who would you like to be there - Who do you need to make peace with - How would you like to be cared for spiritually - What would you like to have happen to your body |

Appendix D

KI Interview guide

| Time | Question/Topic | Prompts |
| --- | --- | --- |
| WARM UP QUESTION: Work Experience with death/dying | | |
|  | Can you tell me a little bit about your background working with homeless or vulnerably housed individuals?  (NOTE: inclusion criteria for study is at least 1 year working with vulnerable population) | Do you have any experiences engaging homeless persons with palliative care?  What situations have influenced your knowledge and understanding of homeless or vulnerably housed clients with palliative care needs? |
| THEME 1: Barriers/Challenges facing homeless people to access palliative care services | | |
|  | Do you think being homeless or vulnerably housed affects a person’s access to palliative care? | what sorts of issues or challenges do homeless individuals face in accessing end-of-life or palliative care? (Get several answers) |
| THEME 2. Does Substance Use affect access to palliative care? | | |
|  | What impact might substance use have on access to palliative care? | Do you believe that an individual's substance use affects their access to end-of-life and palliative care?  How/why? |
| Theme 3: Training/Education/Resources | | |
|  | Do you feel like you have adequate training and resources to support people experiencing homeless and requiring palliative care? | - Yes/no? - If required, prompt: Are you comfortable providing/supporting a homeless or vulnerably housed client in palliative care? - If no, what would make you more comfortable? - What if that person also used substances? |
|  | What education/resources do you think our region needs to engage with people experiences homelessness; homelessness and substance use? |  |
| THEME 4: System Change | | |
|  | What system changes do you think need to happen to help people who are vulnerably housed receive access to palliative care? | What would you like to see change?  What do you think we need to better support vulnerable populations accessing palliative care? |
